# A multi-method exploratory study of health professional students’ experiences with compliance behaviours

**DOI:** 10.1186/s12909-020-02265-4

**Published:** 2020-10-12

**Authors:** Efrem Violato, Sharla King, Okan Bulut

**Affiliations:** Department of Educational Psychology, Faculty of Education, University of Alberta, 6-132 Education North, 11210 - 87 Ave, Edmonton, AB T6G 2G5 Canada

**Keywords:** Compliance, Education, Health, Impression management, Moral distress, Obedience, Conformity

## Abstract

**Background:**

Research in healthcare, including students as participants, has begun to document experiences with negative compliance, specifically conformity and obedience. There is a growing body of experimental and survey literature, however, currently lacking is a direct measure of the frequency at which health professional students have negative experiences with conformity and obedience integrated with psychological factors, the outcomes of negative compliance, and students’ perceptions.

**Methods:**

To develop empirical knowledge about the frequency of negative compliance and student perceptions during health professional education a multi-methods survey approach was used. The survey was administered to health professional students across ten disciplines at four institutions.

**Results:**

The results indicated students regularly experience obedience and conformity and are influenced by impression management and displacement of responsibility. Moral distress was identified as a consistent negative outcome. Student self-reported experiences aligned with the empirical findings.

**Conclusions:**

The findings of the present study demonstrate the pervasiveness of experiences with negative compliance during health professional’s education along with some attendant psychological factors. The findings have educational and practical implications, as well as pointing to the need for further integration of social and cognitive psychology in explaining compliance in healthcare. The results are likely generalizable to a population level however replication is encouraged to better understand the true frequency of negative compliance at a health professional population level.

## Background

Medical errors and patient harm continue to be a problem at all levels of healthcare delivery [1–3] and while the antecedents of harm are complex and span all facets of health systems the causes can generally be categorized as Latent and Active [4]. Active causes of error include those attributable to human actions [4]. Human causes that contribute to harm are widely explored yet there are human factors in need of further elucidation, including group dynamics [5]. A group is three or more people gathered for a common reason whose activity results in some kind of output; the processes, outcomes, and perceptions or experiences of the group are the dynamics [6], see Table 1 for further definitions.

**Table 1:** Common Constructs and Definitions

Construct	Definition	Reference
Compliance	“Compliance refers to a particular kind of response—acquiescence—to a particular kind of communication—a request. The request may be explicit … or it may be implicit.”	(Cialdini & Goldstein, 2004 [7])
Conformity	“Conformity refers to the act of changing one’s behavior to match the responses of others.”	
Obedience	“… behaving in accordance with the opinions, advice, and directives of authority figures”	
Negative Compliance	Potential negative consequences that can arise from deference, yielding or complying with others	(Delaloye, 2017 [8])
	Compliance that produces harm, such as when a person does not speak up or alter a course of action believed to be inaccurate or unsafe	(Green et al., 2017 [9])
Impression Management	“The way people attempt to control the perceptions, or impressions that others have of them, the person’s self-presentation”	(Goffman, 1956 [10])
	“Ways in which people present an image of how they think their audience wishes to see them in face-to-face interaction.”	(Solomon, Solomon, Joseph, & Norton, 2013 [11])
Moral Distress	“The psychological disequilibrium and the state of negative feelings experienced when a person makes a moral decision but does not follow through by performing the moral behaviour indicated by that decision”	(Wilkinson, 1987 [12])
Displacement or Responsibility	“… they [people] view their actions as stemming from the dictates of authorities rather than being personally responsible for them”	(Bandura, 1999 [13])

Within the complexity of group dynamics and possibilities for patient harm, compliance behaviour needs further research [5]. Compliance behaviours can be classed primarily as conformity and obedience. Conformity is behaviour aligning with peers, while obedience is acquiescence to a request made by an authority [7]. When compliance produces harm, such as when a person does not speak up or alter a course of action believed to be inaccurate or unsafe it is considered negative compliance [9]. The challenge for understanding compliance in the context of healthcare is identifying or predicting when harm may occur as conformity and obedience tend to be socially and ecologically adaptive and positive [14, 15]. Much of our learning is social [16] and compliance behaviours are important for learning and understanding norms for appropriate social interactions, learning skills and engaging in accurate behaviour, and self-concept and identity development [17]. As adaptive behaviours conformity and obedience are so functional that it becomes extremely difficult to override them and speak out against an authority or group, to engage in positive deviance, when one thinks what is occurring is wrong [18, 19].

A large degree of the literature published on negative compliance in health care has been focused on compliance in medicine and nursing trainees [20]. Students occupy a low position in healthcare hierarchies and as a result are susceptible to negative compliance through conformity and obedience [8, 21–23]. It is assumed that the issues with negative compliance and the prevailing issues are well documented and understood as negative compliance is becoming more frequently discussed in the literature. However, much of the existing literature covers anecdotal experiences with situations that caused compliance and theoretical suppositions about causes and student experience while lacking causative explanation [24–29]. There is a small but emerging body of experimental work on these constructs based in psychological theory [30–34]. Recent studies have established the high frequency with which medical students and residents, nurses, and staff physicians witness issues with professionalism and patient safety, and experience challenges to speaking up [35–38] yet currently lacking are frequencies of how often health professional students from a range of professions experience negative compliance integrated with psychological theory and relevant constructs [29].

It is not possible to determine these experiences retrospectively through reviews of cases or patient records [39]. Patient records only indicate harm and not the antecedent conditions that led to the outcome. Through observation, it may be possible to see a case where a person should have spoken up but did not. Yet this does not provide information as to why a person did not speak up. Did a person know that something was wrong? Did a person not speak up out of a concern for causing a disturbance? Did the person believe what others were doing was probably correct despite personal uncertainty? The most direct method to understand these questions is to obtain student reports. Without foundational empirical knowledge about the rate of an experience or event integrated with an attempt to understand the social and cognitive aspects of the event, it is not possible to fully understand the phenomenon.

There are numerous variables influencing compliance [7, 8, 40–43]. To understand group dynamics and compliance, it is useful to look at areas outside of the traditional silos of medical knowledge such as psychology [5, 44], which has an extensive history of studying compliance [45]. Three variables that relate to the frequency with which people may engage in compliance that can result in harm are impression management, displacement of responsibility, and moral distress.

Impression management is the way people attempt to control the perceptions, or impressions that others have of them, the person’s self-presentation [10]. Impression management consists of two components, impression motivation and impression construction. Impression motivation is comprised of the goal-relevance, desired outcomes, and discrepancy between the current and desired image. Impression construction consists of the image created in relation to self-concept, desired and undesired identity images, role constraints, target values, and current social image [46]. Expectations of people based on place in a hierarchy can create the conditions for compliance as a person attempts to fulfill the expected role in the context of individual self-presentation, as well as group presentation. Impression Management has been shown to influence compliance behaviours with people being more likely to acquiesce to requests that will present themselves in a positive light to a desired other [47]. For example, in an interprofessional health context nurses in a hospital would engage in impression management to maintain the perception of collaborative work although collaboration was often not possible because of constraints imposed by the practice setting [48]. Expectations regarding different roles have been shown to cause people to behave in accordance with those roles in a professional setting [49].

Displacement of responsibility is identified as one of the most important and strongest influences on submission to authority [13, 50–52]. When a person can or is given the opportunity to displace responsibility for actions or outcomes the person is spared engaging with the outcome and possible rapprochement [13]. One of the primary ways people deal with the displacement of moral control or responsibility is through plausible deniability. The cognitive enactment of plausible deniability is the method through which people engage in motivated reasoning and uncritical acceptance that allows the person to deny they behaved immorally [53] and make themselves believe they did not act immorally [54]. People engage in motivated reasoning for plausible deniability in two ways [55]. First, when considering propositions that people would prefer to be true, they ask “Can I believe this?” Can I believe this has a low evidentiary standard e.g. I did not act immorally because someone else told me to do it. Second, when considering something a person does not want to be true, they will ask “Must I believe this?” Must I believe this has a higher evidentiary standard as some confirmatory evidence is often available e.g. someone became ill because I did what I was told, and requires a more rigorous search for a reason not to believe it e.g. if I didn’t do what I was told I would have failed placement and someone else would have done it anyways. The influence of displacement of responsibility has been demonstrated in healthcare [50] and numerous other areas (Bandura [13]; Davis et al. [51]; Meeus & Raaijmakers [56]; Richardot [57]).

Negative personal outcomes can occur because of compliance, whether compliance occurred through impression management, displacement of responsibility, or other means. A highly impactful negative outcome is Moral Distress. Jameton [58] defined moral distress as: “negative feelings that arise when one knows the morally correct response to a situation but cannot act accordingly because of institutional or hierarchical constraints.” Wilkinson [12] further accounts for the psychological factors of distress “the psychological disequilibrium and the state of negative feelings experienced when a person makes a moral decision but does not follow through by performing the moral behaviour indicated by that decision”. Moral Distress can arise in healthcare due to conflict that occurs between the maintenance of a person’s moral integrity, the internal consistency of a set of personal standards, and behaviour constrained by external factors [59]; or more simply the experiencing of a moral event and the resultant psychological distress [60]. Institutional, environmental or system factors can create moral distress through challenges imposed to the maintenance of moral integrity creating a sense of futility and can lead to negative psychological and physical outcomes including burnout, fatigue, disengagement and increased susceptibility to negative compliance [61–65].

The principles of compliance are nearly universal [66–70] and function similarly in healthcare as in other contexts. Obtaining insight into how students experience and think about compliance and in what ways these are related to, or reflect, frequency of behaviour can aid in developing a better understanding of where more detailed research is needed and what means might be taken to address negative compliance. It is necessary to know the extent of the problem and why it occurs. Is negative compliance something only rarely experienced but because of its impact it is highly salient and frequently discussed or is it something experienced daily and the risk of harm is even higher than anticipated? Does impression management factor into compliance or is it strictly a matter of displacement of responsibility? These things are unknown.

The present study will take an exploratory approach to understand the frequency with which students experience negative compliance related to obedience and conformity, and three related psychological phenomena. Two research questions have been developed utilizing a multi-method approach to examine experiences and cognitions around compliance behaviour.

### Research questions

1. *Experiences and Expected Behaviour*: How frequently do students experience negative compliance through conformity and obedience and what are some of the possible underlying social and psychological influences and outcomes?
2. *Perceptions of Compliance*: What are students’ perceptions of obedience and conformity? Can common themes be derived from these?

## Methods

### Survey development

A self-reported survey with 39 questions was designed to explore the frequencies of participants’ experiences with Conformity and Obedience including Impression Management, Diffusion of Responsibility, and Moral Distress. Included as a part of the survey was a separate measure to examine the Better than Average Effect. Results of the BTAE portion of the survey will be reported in a separate publication. Survey items were tested using cognitive interviews [71] conducted with practitioners and students from the fields of health sciences, psychology, educational psychology, and business. Items were modified based on feedback provided during the cognitive interviews.

### Analysis

All statistical analyses were carried out using SPSS Version 25 [72]. Frequencies and descriptive statistics were used for the analysis of rates of behaviour. Open-ended responses were independently examined for general themes by each of the authors. The authors then compared the independently identified themes to identify commonalities and discrepancies.

### Participants

Participants were recruited from ten health sciences programs from four institutions with credentials ranging from certificate programs to graduate degrees. Participants were recruited to the study through emails sent through departmental listservs at the University of Alberta and during an interprofessional simulation event hosted at the University of Alberta during the 2019 Winter Semester. The survey was completed online through the web-based survey platform Qualtrics. Participants reviewed and completed a consent form, providing written consent, prior to completion of the survey. Ethics approval was granted by Research Ethics Board 2 at the University of Alberta, Pro00081948.

A total of 102 participants began the survey with 69 completing the entire survey. Data were examined for outliers, two influential cases were identified and determined to be careless responding and so were removed from the dataset (Table 2).

**Table 2:** Sample Demographic Information

Type of Institution	Program	n (% of sample)	Sex	Age Mean (SD)	PGY Mean (SD)
University Undergraduate	KSR	7 (10%)	F = 5 M = 2	21.71 (2.72)	2.57 (1.40)
	Pharmacy	5 (7.4%)	F = 2 M = 1	22.28 (2.91)	2.00 (1.15)
	Medicine	5 (7.4%)	F = 4 M = 1	26.31 (3.95)	1.20 (.989)
	ALES	5 (7.4%)	F = 4 M = 1	21.3 (1.65)	3.50 (.87)
	Nursing	2 (3%)	F = 2 M = 0	20.5 (0.51)	1.00 (X)
	Social Work	1 (1.4%)	F = 1 M = 0	24.0 (X)	1.00 (X)
University Graduate	RM	30 (45%)	F = 25 M = 5	25.2 (2.90)	1.08 (.475)
Polytechnical	RT	6 (9%)	F = 5 M = 1	22.67 (1.87)	2.5 (.77)
	ACP	1 (1.4%)	F = 0 M = 1	26.0 (X)	1.00 (X)
College	PharmTech	5 (7.4%)	F = 5 M = 0	21.01 (1.10)	1.5 (.50)
Total		*N* = 67	F = 55 M = 12	23.79 (3.1)	1.83 (1.20)
				Range = 19–33	

*KSR* Kinesiology Sport and Recreation, *ALES* Agriculture Life and Environmental Sciences, *RM* Rehabilitation Medicine, *RT* Respiratory Therapy, *ACP* Advanced Care Paramedic, *PharmTech* Pharmacy Technician

## Results

### Experiences, influences, and outcomes

On average, in the last week during training participants reported experiencing situations where they conformed to peers behaviour 3.2 times and were obedient to an authority 2.3 times (Table 3).

**Table 3:** Mean number of experiences with conformity and obedience

Time Frame	Conformity		Obedience	
	Self	Peer	Self	Peer
Past Week	3.2 (2.2)	4.83 (2.74)	2.3 (2.5)	2.57 (2.4)
Past Month	8.11 (4.96)	10.63 (5.7)	4.33 (3.9)	5.39 (4.0)
Past Six Months	23.5 (12.83)	26.75 (17.53)	11.77 (11.79)	16.2 (12.96)

Note: Standard deviations are shown in parentheses

The majority of the sample (84.6%) indicated they had carried out a task in the way a peer did when uncertain of the proper method; participants indicated they had often observed peers do the same (91%). When performing a procedure or technique participants felt peers frequently went with the crowd (53%) at a higher rate than themselves (27%), most participants (60%) believed they only sometimes went with the crowd. Participants tended to feel somewhat confident in their knowledge when a peer disagreed with them (79%), a minority (6%) were completely confident.

Almost all participants (91%) had been in a situation where they followed the instruction of an authority feeling that they could not contradict the person despite believing the person in authority to be incorrect. An equal number had a peer disclose being in a similar situation. Most participants (81%) indicated they had followed peers that were acting on instruction from an authority that the participant did not believe to be correct. Fifty-one percent of participants had been subjected to negative consequences for speaking up and 65% had witnessed peers being subjected to negative consequences for speaking up.

Participants indicated Impression Management was a factor for obedience and conformity. Sixty-nine percent of participants had acted on incorrect instructions from an authority because of concern with how the authority would perceive them personally, with 68% reporting concern with how they would be perceived professionally. Participants indicated a high level of concern with being viewed as a typical member of their profession (60%) yet to a lesser degree indicated a high level of concern with how peers would view them professionally (35%), a small number (10%) were never concerned with how others thought of them. Most participants felt it necessary to alter their behaviour (89%) and thinking (67%) to align with the behaviour and thinking of those around them. Almost all participants felt a need to “fake it until you make it” (94%) with many feeling that they often needed to do so (37%).

Displacement of Responsibility was frequently indicated as a factor in obedience with 66% of participants having followed the instructions of an authority because they did not believe that they themselves would be held personally responsible for the outcome.

Being obedient to an authority caused Moral Distress for participants. Acting on the instructions of an authority believed to be incorrect had caused distress for most participants (71%), with 51% having felt highly distressed and 13% extremely distressed. Similar distress was witnessed in peers (73%), with participants reporting peers had indicated they had been highly distressed (53%) and extremely distressed (6%).

### Perceptions of compliance

Responses to the open-ended item provide further insight into participant experiences. Four primary themes were identified in the responses: Desire for Smooth Interactions, Student-Instructor Dynamic, Experience and Knowledge as Supportive Factors, and Need for Education on Positive Deviance.

#### Desire for smooth interactions

Several of the participants stated that the avoidance of conflict and a desire for smooth interactions was a central reason to obey authority figures. Some participants stated that they often go along with what the instructor says for the purposes of assessment. Participants behavior or actions would adapt for the clinical environment, but it is not ‘worth it’ to them to question their instructor’s actions.

*“I think that sometimes we may go with what an instructor says just because that is the way we are supposed to do it for the purposes of the course. Rather than speak out against what we think might not be the best way of doing something, we just learn it for the test and know we won't actually do it that way in practice”*

(20–25-year-old, Occupational Therapy Student)

*“One reason that I obey authority figures is that I do not want to start a loud argument or 'create a scene’”*

(20–25-year-old, Dietetics Student)

#### Student-instructor dynamic

Participants mentioned the nature of the student-instructor relationship creates a power differential that creates discomfort with questioning authority.

*“I find that power dynamics (e.g. Those between a senior faculty member and a student) make it very difficult to feel comfortable or able to question the authority figure. Conformity is often very hard to avoid in that sense, I have found.”*

(20–25-year-old, Kinesiology Student)

#### Experience and knowledge as supportive factors

Students believed more experience and knowledge were a means to counterbalance obedience to authority. Having the confidence and comfort level to speak up and ask questions within a specific context was mentioned.

*“I feel like a lot of my obedience was in years past and now I do feel more comfortable asking questions and clarifying before I do something. This is typically based on experience in the area.”*

(26–30-year-old, Occupational Therapy Student)

#### Need for education on positive deviance

A few participants expressed a need for education on dealing with situations of obedience, two forms of need were identified. First, participants commented that teaching students how to engage instructors who don’t want to hear opposing ideas would better prepare students to manage situations where they need to question authority in potentially conflictual situations. Second, participants desire faculty development and education for instructors about positive deviance and the need to encourage questions and opposing beliefs from students to counteract obedience and create a learning space that allows for open discussion.

*“It is difficult to say no or to oppose the beliefs of instructors that do not know how to handle hearing what they don't want to hear. More needs to be done to educate students on how to handle these conflicts.”*

(20–25-year-old, Physiotherapy Student)

The participants written comments indicate that students have experienced issues of obedience in the educational setting yet have found strategies to avoid the situation when they did arise. With time (experience) and greater knowledge, they felt the frequency and/or impact of these situations would lessen.

## Discussion

The results of the two research questions are integrative and supportive, with a general picture of compliance behaviour emerging. Within a health sciences education context, experiences with obedience and conformity are common and students are aware of some of the causes of compliance, yet often do not think that it is possible to engage in positive deviance.

### Experiences, influences, and outcomes

Almost all participants had experienced conformity and obedience in their training. Most participants indicated Displacement of Responsibility was a factor in obedience, having acted on the instructions of an authority because they did not believe that they would be held personally responsible, aligning with the findings of other research [8, 25, 50]. Though displacement of responsibility can be legally exonerating [73, 74] there is an additional moral responsibility beyond the legal one for people to engage in positive deviance when it is believed something inappropriate is occurring [63]. Further, when students displace responsibility and are obedient it can have a negative personal impact, namely Moral Distress, with its attendant negative outcomes. Interestingly the reported experiences with obedience and conformity decreased as the time frame of recall increased. It is possible students may simply be unable to recall events as accurately as the time frame increases. Forgetting may also be a protective mechanism against Moral Distress, further investigation into a possible effect is merited. The desire for smooth social interactions through Impression Management was also prevalent. The results from the survey items and common themes identified in the qualitative analysis identify Impression Management as an important factor in eliciting compliance in health professional students. The desire for smooth interaction can stem from concerns over the result of speaking up, or non-obedience, including ostracization, punishment, or being labelled negatively by those more advanced in a hierarchy, though reproach can also come from peers [75]. The prevalence of Displacement of Responsibility and Impression Management has important implications for patient safety. It is necessary to instill in learners the shared responsibility of all team members, from students to senior practitioners, for patient outcomes and that patient outcomes are ultimately more important than transient interpersonal aspects of a healthcare team. Establishing shared responsibility is important in the delivery of patient-centered care and avoiding negative compliance and engaging in positive deviance could have the benefit of preventing Moral Distress.

### Perceptions of compliance

The themes identified from the student comments helped to elucidate the results of the first research question. Though students may believe that something is wrong, they still engage in Impression Management as there is a *Desire for Smooth Interactions* by avoiding conflict. The desire to avoid conflict may depend on the personality of the student and instructors, the context of the situation, and potential future assessment. The avoidance of conflict is also related to completing curricular requirements and learning what is necessary for assessment. To an extent, compliance is necessary for education and students are willing to forgo conflict to learn required knowledge. The survey items indicating a concern for the perceptions of others and a need to be viewed as a competent, typical member of the profession are supported by the reports of a desire for smooth interaction.

A student’s level of *Experience and Knowledge as Supportive Factors* can facilitate students speaking up in situations when they observe something is wrong. As a person moves through their education and becomes more certain in their knowledge and their position in a health care team the person can feel more confident in challenging an authority. The validity of students’ belief that experience will result in better performance is questionable when considering other research indicating compliance is prevalent across all levels of age and experience and expected behaviour is a poor predictor of actual behaviour [54, 76–82]. The potential protective effect of experience and knowledge requires further empirical examination.

Participants may want to speak up but have a *Need for Education on Positive Deviance* to help them do so. The need for personal education extends to a desire for education for those in positions of authority to help the authority to be open to challenges. Students believe early instruction on compliance and positive deviance could be beneficial for when students are in future positions of authority so that they will not be as rigid as predecessors and will engage in open discussion with those in a lower hierarchical position. Education on these topics could be further bolstered by teaching Social and Cognitive Psychology in the health sciences curriculum, potentially in interprofessional courses [83, 84].

### Educational implications

The frequency of compliance has educational implications that could influence educational interventions targeted at reducing negative compliance and increasing positive deviance. In the healthcare literature there is growing attention to the fact a large part of a student’s education, informally, is through the Hidden Curriculum [19, 85, 86]. One of the key components of identifying, understanding, and conveying knowledge about the Hidden Curriculum to students is through formal educational means. Formal education that addresses the Hidden Curriculum includes providing information on specific outcomes that result from the Hidden Curriculum including compliance behaviour, how to identify situations of negative compliance and how to engage in positive deviance [19, 87]. Patient advocacy tools for engaging in positive deviance, such as the Advocacy-Inquiry approach, CUS, and the two-challenge rule, and programs such as TeamSTEPPS, could be taught in conjunction with information regarding the Hidden Curriculum and the social and cognitive psychological causes of compliance [88–90]. A more thorough understanding of why it is difficult to engage in positive deviance and speak up may make the programs more effective and the tools easier to use.

People are predisposed to obedience to authority and group loyalty [70, 91] yet believe that they are not influenced by either [92, 93]. Part of education on the Hidden Curriculum should emphasize the frequencies of experiences and that all students will experience compliance scenarios and with a high degree of likelihood they will be compliant. More specifically, students should be informed of base rates and that despite a belief that they will be in the minority that speaks up, it is statistically impossible [94]. There are also implications for educators and administrators, it is necessary to make those in advanced hierarchical positions aware of how their actions have behavioural and psychological impacts on students. Increased awareness can be combined with recommendations for educators and administrators such as respecting and even requesting dissenting opinions. Leader inclusiveness demonstrating invitation and appreciation through words and deeds in an interdependent setting can promote psychological safety regardless of role status [95]. The promotion of psychological safety can help change the Hidden Curriculum, and subsequently broader institutional culture, to create safe and supportive environments for positive deviance.

### Limitations

There are two main limitations to the present study. First, there is a small sample size. Improved sample coverage would increase the validity of the interpretations of the frequencies of compliance in the population of health sciences students. Additionally, it is possible there was a response bias with students particularly sensitive to the issues being more likely to complete the survey. With the recognition of the sample size, the nature of the study is exploratory and was able to capture a diversity of professions in programs that range from certificates to graduate degrees from multiple institutions. Many of these disciplines have no research on compliance and the present study serves as an initial exploration into conformity and obedience for these disciplines. Furthermore, the results of the present research align with anecdotal evidence and experimental findings [24, 25, 31, 50, 96–100]. Second, a related limitation is a large number of participants were from the Rehabilitation Medicine (RM) program. It might be argued the results are primarily representative of RM, however, RM does cover several subdisciplines and no differences were found across any of the programs or institutions for any of the survey measures. The fundamental constructs of human behaviour are nearly universal, including compliance, and it is highly likely that the results of the present study will generalize to other samples and populations and similar results would be found [66–70, 101]. The authors encourage broad multi-institutional replication to verify the generalizability of these findings (see Supplemental Material for a copy of the survey).

## Conclusions

It was found that situations of obedience and conformity are commonly experienced across health science programs. The present study contributes to the existing literature by providing a measure of experiences of negative compliance integrated with relevant psychological theories. The present results provide further empirical support to the existing literature on negative compliance as well as why it occurs. Educational implications can be derived from this work including the need to make students aware of the likelihood that there is a tendency to be compliant in challenging scenarios despite personal expectations to the contrary. It was also identified that students desire more education on compliance and positive deviance.

The present study is an example of the integration of causative mechanisms with sampling of negative compliance and the challenge of speaking up. It is necessary for widespread replication of the present research with expanded sampling to determine the rates of conformity and obedience, as well as the attendant psychological variables, at the population level of health professional students in North America and globally. Extension should also include sampling of instructors and preceptor’s knowledge and experiences of compliance. More data will produce normative rates and will allow contrast and comparison to help determine if any single institution or program has an exceptional issue with negative compliance and evaluate the efficaciousness of educational programs in improving positive deviance.

## Supplementary information

**Additional file 1.** Compliance Behaviour Survey

## Data Availability

The datasets generated during and/or analyzed during the current study are not publicly available due the conditions of the University of Alberta Research Ethics Boards approval for the study. Data are available from the corresponding author on reasonable request and approval from the University of Alberta REB.

## Abbreviations

BTAE: Better than Average Effect
SPSS: Statistical Package for the Social Sciences
REB: Research Ethics Board
TeamSTEPPS: Team Strategies and Tools to Enhance Performance and Patient Safety
KSR: Kinesiology Sport and Recreation
ALES: Agriculture Life and Environmental Sciences
RM: Rehabilitation Medicine
RT: Respiratory Therapy
ACP: Advanced Care Paramedic
PharmTech: Pharmacy Technician

## References

[1] Canadian Institute for Health Information (2019). Hospital Harm Results 2014–2015 to 2018–2019.

[2] Makary MA, Daniel M (2016). Medical error — the third leading cause of death in the US. BMJ.

[3] Vogel L (2016). One in 18 patients harmed in hospital. CMAJ..

[4] Kohn LT, Corrigan JM, Donaldson MS (1999). To err is human: building a safer health system [internet]. Institute of Medicine. Institute of Medicine.

[5] Kaba A, Wishart I, Fraser K, Coderre S, Mclaughlin K (2016). Are we at risk of groupthink in our approach to teamwork interventions in health care?. Med Educ.

[6] Tasca GA (2020). What is group dynamics? Gr Dyn theory. Res Pract.

[7] Cialdini RB, Goldstein NJ (2004). Social influence: compliance and conformity. Annu Rev Psychol.

[8] Delaloye NJ. An Exploration of Deference Behaviours Exhibited within the Paediatric Resuscitation Environment (Unpublished master's thesis). Calgary: University of Calgary; 2017. 10.11575/PRISM/24890.

[9] Green B, Oeppen RS, Smith DW, Brennan PA (2017). Challenging hierarchy in healthcare teams – ways to flatten gradients to improve teamwork and patient care. Br J Oral Maxillofac Surg.

[10] Goffman E (1956). The presentation of self in everyday life.

[11] Solomon JF, Solomon A, Joseph NL, Norton SD (2013). Impression management, myth creation and fabrication in private social and environmental reporting: insights from Erving Goffman. Account Organ Soc.

[12] Wilkinson JM (1987). Moral distress in nursing practice: experience and effect. Nurs Forum.

[13] Bandura A (1999). Moral disengagement in the perpetration of inhumanities. Personal Soc Psychol Rev.

[14] Marsh B, Todd PM, Gigerenzer G, Leighton JP, Sternberg RJ (2004). Cogntive heuristics: reasoning the fast and frugal way. The nature of reasoning.

[15] Todd PM, Gigerenzer G, Dennis WM, Katsikopoulos KV, Goldstein DG, Dieckmann A, Berg N (2012). Ecological rationality: intelligence in the world. Designed to fit minds.

[16] Bandura A (1971). Social learning theory.

[17] Cialdini RB, Trost MR, Fiske ST, Lindzey G (1998). Social influence: social norms, conformity, and compliance. The Handook of social psychology.

[18] Cialdini RB (2006). Influence: the psychology of persuasion.

[19] Holmes CL, Harris IB, Schwartz AJ, Regehr G (2014). Harnessing the hidden curriculum: a four-step approach to developing and reinforcing reflective competencies in medical clinical clerkship. Adv Heal Sci Educ..

[20] Kilpatrick K, Paquette L, Jabbour M, Tchouaket E, Fernandez N, Al Hakim G, et al. Systematic review of the characteristics of brief team interventionsto clarify roles and improve functioning in healthcare teams. PLoS One. 2020;15(6):e0234416. 10.1371/journal.pone.0234416 PMC7286504 32520943

[21] Coombs M (2003). Power and conflict in intensive care clinical decision making. Intensive Crit Care Nurs.

[22] Sexton JB, Thomas EJ, Helmreich RL, Bmj S, Medical B, Mar N (2015). BMJ error , Stress , and Teamwork in Medicine and Aviation: Cross Sectional Surveys. BMJ.

[23] Delaloye NJ, Tobler K, O’ neill T, Kotsakis A, Cooper J, Bank I (2017). Errors during resuscitation: the impact of perceived authority on delivery of care. J Patient Saf.

[24] Liao JM, Thomas EJ, Bell SK (2014). Speaking up about the dangers of the hidden curriculum. Health Aff.

[25] Sur MD, Schindler N, Singh P, Angelos P, Langerman A (2016). Young surgeons on speaking up: when and how surgical trainees voice concerns about supervisors’ clinical decisions. Am J Surg.

[26] Wray T, Yu C, Philbey C, Salloch S, Sandow V, Schildmann J, Vollmann J (2016). Akrasia and obedience in medicine: deferring to authority in a situation you believe to be wrong. Ethics and professionalism in healthcare: transition and challenges.

[27] Voogt JJ, Kars MC, van Rensen ELJ, Schneider MME, Noordegraaf M, van der Schaaf MF. Why Medical residents do (and Don’t) speak up aboutorganizational barriers and opportunities to improve the quality of care. Acad Med. 2020;95(4):574-81. 10.1097/ACM.0000000000003014 31577591

[28] Fisher M, Kiernan M (2019). Student nurses’ lived experience of patient safety and raising concerns. Nurse Educ Today.

[29] Hurley J, Hutchinson M, Peadon R (Rod) (2020). Hierarchy and medical error: Speaking up when witnessing an error. Saf Sci.

[30] Kaba A, Beran TN (2016). Impact of peer pressure on accuracy of reporting vital signs: an interprofessional comparison between nursing and medical students. J Interprof Care..

[31] Beran TN, McLaughlin K, Al Ansari A, Kassam A (2013). Conformity of behaviors among medical students: impact on performance of knee arthrocentesis in simulation. Adv Heal Sci Educ.

[32] Beran T (2015). Research advances in conformity to peer pressure: a negative side effect of Medical education. Heal Prof Educ.

[33] Daly Guris RJ, Duarte SS, Miller CR, Schiavi A, Toy S (2019). Training novice anaesthesiology trainees to speak up for patient safety. Br J Anaesth.

[34] Kuo SY, Wu JC, Chen HW, Chen CJ, Hu SH (2020). Comparison of the effects of simulation training and problem-based scenarios on the improvement of graduating nursing students to speak up about medication errors: a quasi-experimental study. Nurse Educ Today.

[35] Schwappach D, Sendlhofer G, Kamolz LP, Köle W, Brunner G (2019). Speaking up culture of medical students within an academic teaching hospital: need of faculty working in patient safety. PLoS One.

[36] Mak-van der Vossen M, Teherani A, van Mook WNKA, Croiset G, Kusurkar RA (2018). Investigating US medical students’ motivation to respond to lapses in professionalism. Med Educ.

[37] Martinez W, Lehmann LS, Thomas EJ, Etchegaray JM, Shelburne JT, Hickson GB (2017). Speaking up about traditional and professionalism-related patient safety threats: a national survey of interns and residents. BMJ Qual Saf.

[38] Schwappach D, Sendlhofer G (2020). Speaking up about patient safety in perioperative care: differences between academic and nonacademic hospitals in Austria and Switzerland. J Investig Surg.

[39] Norman GR, Monteiro SD, Sherbino J, Ilgen JS, Schmidt HG, Mamede S (2017). The causes of errors in clinical reasoning: cognitive biases, knowledge deficits, and dual process thinking. Acad Med.

[40] Bègue L, Beauvois JL, Courbet D, Oberlé D, Lepage J, Duke AA (2015). Personality predicts obedience in a Milgram paradigm. J Pers.

[41] Blass T (1999). The Milgram paradigm after 35 years: some things we now know about obedience to authority. J Appl Psychol.

[42] Lepage J, Bègue L, Zerhouni O, Courset R, Mermillod M (2018). Influence of authoritarianism, vagal tone and mental fatigue on obedience to authority. Cogn Emot.

[43] Milgram S (1965). Some conditions of obedience and disobedience to authority. Hum Relations.

[44] Croskerry P, Cosby KS, Graber ML, Singh H (2017). Diagnosis: interperting the shadows.

[45] Reis HT, Baumeister R, Finkel EJ (2010). How we got from Here to there: a brief history of social psychology. Advanced social psychology: state of the science.

[46] Leary MR, Kowalski RM (1990). Impression Managment: a literature review and two-component model. Psychol Bull.

[47] Rind B, Benjamin D (1994). Effects of Public Image Concerns and Self-image on Compliance. J Soc Psychol.

[48] Lewin S, Reeves S (2011). Enacting “team” and “teamwork”: using Goffman’s theory of impression management to illuminate interprofessional practice on hospital wards. Soc Sci Med.

[49] Guadagno RE, Cialdini RB (2007). Gender differences in impression management in organizations: a qualitative review. Sex Roles.

[50] Bould MD, Sutherland S, Sydor DT, Naik V, Friedman Z (2015). Residents’ reluctance to challenge negative hierarchy in the operating room: a qualitative study. Can J Anesth Can d’anesthésie.

[51] Davis S, DeZoort FT, Kopp LS (2006). The effect of obedience pressure and perceived responsibility on management accountants’ creation of budgetary slack. Behav Res Account.

[52] Milgram S (1963). Behavioral study of obedience. J Abnorm Soc Psychol.

[53] Bersoff D (1999). Why good people sometimes do bad things: motivated reasoning and unethical behavior. Personal Soc Psychol Bull.

[54] Ariely D (2008). Predictably irrational: the hidden forces that shape our decisions.

[55] Epley N, Gilovich T (2016). The mechanics of motivated reasoning. J Econ Perspect.

[56] Meeus WHJ, Raaijmakers QAW (1986). Administrative obedience: carrying out orders to use psychological-administrative violence. Eur J Soc Psychol.

[57] Richardot S (2014). “You know what to do with them”: the formulation of orders and engagement in war crimes. Aggress Violent Behav.

[58] Jameton A (1984). Nursing practice: the ethical issues.

[59] de Raeve L (1998). Maintaining integrity through clinical supervision. Nurs Ethics.

[60] Morley G, Ives J, Bradbury-Jones C, Irvine F (2019). What is “moral distress”? A narrative synthesis of the literature. Nurs Ethics.

[61] Atabay G, Çangarli BG, Penbek Ş (2015). Impact of ethical climate on moral distress revisited: multidimensional view. Nurs Ethics.

[62] McAndrew NS, Leske J, Schroeter K (2018). Moral distress in critical care nursing: the state of the science. Nurs Ethics.

[63] Monrouxe L, Shaw M, Rees C (2017). Antecedents and consequences of Medical students’ moral decision making during professionalism dilemmas. AMA J Ethics.

[64] Schwenzer KJ, Wang L (2006). Assessing moral distress in respiratory care practitioners. Crit Care Med.

[65] Wiggleton C, Petrusa E, Loomis K, Tarpley J, Tarpley M, O’Gorman M (2010). Lou, et al. Medical students’ experiences of moral distress: development of a web-based survey. Acad Med.

[66] Greene JD, Decety J, Wheatley T (2015). The cognitive neuroscience of moral judgment and decision making. The moral brain: a multidisciplinary perspective.

[67] Graham J, Nosek BA, Haidt J, Iyer R, Koleva S, Ditto PH (2011). Mapping the moral domain. J Pers Soc Psychol.

[68] Haidt J (2007). The new synthesis in moral psychology. Science..

[69] Avorn J (2018). The psychology of clinical decision making - Implications for medication use. New Eng J Med.

[70] Haidt J (2012). The righteous mind: why good people are divided by politics and Relgion.

[71] Peterson CH, Peterson NA, Powell KG (2017). Cognitive interviewing for item development: validity evidence based on content and response processes. Meas Eval Couns Dev.

[72] IBM Corp (2017). IBM SPSS statistics for windows.

[73] Darley JM (1995). Constructive and destructive obedience: a taxonomy of principal-agent relationships. J Soc Issues.

[74] Hamilton V, Sanders J (1995). Crimes of obedience and conformity in the workplace: surveys of Americans, Russians, and Japanese. J Soc Issues.

[75] Blenkinsopp J, Snowden N, Mannion R, Powell M, Davies H, Millar R (2019). Whistleblowing over patient safety and care quality: a review of the literature. J Health Org Manag.

[76] Calhoun AW, Boone MC, Miller KH, Pian-Smith MCM (2013). Case and commentary: using simulation to address hierarchy issues during medical crises. Simul Healthc.

[77] Harley EM, Carlsen KA, Loftus GR (2004). The “ Saw-It-All-Along ” Effect : Demonstrations of Visual Hindsight Bias. J Exp Psychol Learn Mem Cogn.

[78] Perugini M, Leone L (2009). Implicit self-concept and moral action. J Res Pers.

[79] Pieters R, Baumgartner H, Bagozzi R (2006). Biased memory for prior decision making : Evidence from a longitudinal field study. Org Behav Hum Dec Process.

[80] Shafir E, Leboeuf RA (2002). Rationality. Annu Rev Psychol.

[81] Sunstein CR, Thaler RH (2003). Libertarian paternalism is not and oxymoron. Univ Chicago Law Rev.

[82] Zell E, Krizan Z (2014). Do people have insight into their abilities? A Metasynthesis. Perspect Psychol Sci.

[83] Royce CS, Hayes MM, Schwartzstein RM (2019). Teaching critical thinking: a case for instruction in cognitive biases to reduce diagnostic errors and improve patient safety. Acad Med.

[84] Sussman JD (2019). Benefits of heuristic and Bias awareness in Medical education. Acad Med.

[85] Hafferty FW, Castellani B. The hidden curriculum: a theory of medical education. Routledge: Handbook of the sociology of medical education; 2009. p. 29–49.

[86] Raso A, Marchetti A, D’Angelo D, Albanesi B, Garrino L, Dimonte V (2019). The hidden curriculum in nursing education: a scoping study. Med Educ.

[87] Kumar K, Silverberg S, Sud A (2018). The Hidden Curriculum: A Primer for Canadian Medical Students. Canadian Federation of Medical Students.

[88] Clapper TC (2018). TeamSTEPPS ® is an effective tool to level the hierarchy in healthcare communication by empowering all stakeholders. J Commun Healthcare.

[89] Omura M, Maguire J, Levett-Jones T, Stone TE (2017). The effectiveness of assertiveness communication training programs for healthcare professionals and students: a systematic review. Int J Nurs Stud.

[90] Chen AS, Yau B, Revere L, Swails J (2019). Implementation, evaluation, and outcome of TeamSTEPPS in interprofessional education: a scoping review. J Interprof Care.

[91] Haidt J (2001). The emotional dog and its rational tail: a social intuitionist approach to moral judgment. Psychol Rev.

[92] Doliński D, Grzyb T, Folwarczny M, Grzybała P, Krzyszycha K, Martynowska K (2017). Would you deliver an electric shock in 2015? Obedience in the experimental paradigm developed by Stanley Milgram in the 50 years following the original studies. Soc Psychol Personal Sci.

[93] Grzyb T, Dolinski D. Beliefs about obedience levels in studies conducted within the milgram paradigm: better than average effect and comparisons of typical behaviors by residents of various nations. Front Psychol. 2017;8. 10.3389/fpsyg.2017.01632. 10.3389/fpsyg.2017.01632 PMC5611685 28979232

[94] Zell E, Strickhouser JE, Sedikides C, Alicke MD (2019). The better-than-average effect in comparative self- evaluation: a comprehensive review and meta-analysis. Psychol Bull.

[95] Nembhard IM, Edmondson AC (2006). Making it safe: the effects of leader inclusiveness and professional status on psychological safety and improvement efforts in health care teams. J Organ Behav.

[96] Beran T, Kaba A, Caird J, McLaughlin K (2014). The good and bad of group conformity: a call for a new programme of research in medical education. Med Educ.

[97] Calhoun A (2014). Using simulation to address hierarchy-related errors in Medical practice. Perm J.

[98] Cosby KS, Croskerry P (2004). Profiles in patient safety: authority gradients in medical error. Acad Emerg Med.

[99] Kaba A, Beran TN, White D (2016). Accuracy of interpreting vital signs in simulation: an empirical study of conformity between medical and nursing students. J Interprofessional Educ Pract.

[100] Sydor DT, Bould MD, Naik VN, Burjorjee J, Arzola C, Hayter M (2013). Challenging authority during a life-threatening crisis: the effect of operating theatre hierarchy. Br J Anaesth.

[101] Pinker S (2002). The blank slate: the modern denial of human nature.

