# Supplementary material for: A multi-method exploratory study of health professional students’ experiences with compliance behaviours
Source: BMC Med Educ. 2020 Oct 12;20:359. doi: 10.1186/s12909-020-02265-4 (PMC7552343; doi:10.1186/s12909-020-02265-4)
Supplement: Supplementary file 1 — Additional file 1. Compliance Behaviour Survey [file 12909_2020_2265_MOESM1_ESM.docx]

**Compliance Behaviour Survey**

*Introduction*: Throughout this survey you will be presented with questions based on your experiences, your perceptions and some demographic information. For each question please select the answer that you feel is most applicable to you or best represents your experiences. You may decline to answer a particular question, in this case leave the question blank and complete the questions you feel comfortable answering.

____________________________________________________________________________

In the first section of this survey we will present you with four brief stories. Please read each story carefully and then respond to the questions.

Story 1: Tom is taking a workshop about how to treat mild burns. During a portion of the workshop there is a simulated case study session with a mannequin where an instructor, who is an emergency room doctor, provides one on one advice on how to treat different burn cases. The simulation case Tom has been assigned to is a first aid situation that involves a burn on a person’s forearm that has begun to blister. The blister has swollen to a large size and Tom is trying to apply a clean bandage, but the blister is making it difficult to apply the bandage. The instructor watches Tom struggle and then advises him to “break the blister” so that it will be easier to apply the bandage. From the pre-course material that Tom read and from a previous CPR course Tom took he knows that you should never break a blister when providing first aid. Tom responds that he does not think this is correct. The instructor responds in a clear voice that Tom should break the blister. Tom follows the instructor’s directions and breaks the blister.

Story 2: Steven was with a group of three other students from his class. The group was learning how to take a Resting Pulse and Resting Respiratory Rate from a patient. Before beginning to take measurements, the instructor told the group how important it was to take accurate readings as these would impact the care a patient receives. As each student took turns measuring the Resting Pulse and Resting Respiratory Rate for the patient they were asked to state each measure out loud so their instructor could record the results and verify if they had taken the correct measurements. Nicole went first and read out the resting pulse as 56, and respiratory rate as 22, the instructor marked down the readings. Dallas went second and indicated the resting pulse was 58 and the respiratory rate was 24. Li was third and stated the resting pulse was 58 and the respiratory rate was 22. Steven was last and as he took the readings from the patient he obtained a resting pulse of 70 and a respiratory rate of 17, unsure of his measurements he took them again and received the same numbers. Steven then stated to the instructor that the resting pulse was 57 and the respiratory rate was 23.

Story 3: Jane is in a basic anatomy class. In this class the teacher likes to call on students to provide answers, the teacher will often call a student by name to answer a question. At the beginning of each class the teacher reviews material from the previous class. The previous class was regarding the muscles of the leg. As the teacher is going through the muscles of the leg the teacher asks several students in a row to indicate the proper anatomical structure of a muscle before indicating if the students answer was correct or not. The teacher asks Brad, Kat and Jane where the insertion of the biceps femoris is. Brad responds first that it is the head of the *tibia*, Kat replies next and with confidence gives the same answer as Brad. To Jane, Brad and Kats answer doesn’t sound quite right, she is fairly certain the insertion is at the head of the *fibula*. Jane has paused for a second and the teacher is looking at her, so she gives the same answer as Brad and Kat.

Story 4: Natalie is a health sciences student working as a caregiver at an assisted living facility during the summer. When Natalie first started at the assisted living facility she was mostly helping the residents get around, making sure they were comfortable and ensuring that they received their meals. Since Natalie had shown herself to be very responsible the nurses at the facility have been giving her more and more responsibilities. In the last couple of weeks, she had started administering medication to patients. One day while Natalie was working at the front desk of the nursing home a call came in. The call was from a person identifying themselves as Dr. Campbell, Natalie had never met a Dr. Campbell while working at the assisted living facility. Dr. Campbell stated that he was the physician assigned to the facility and he was based at a local clinic. Dr. Campbell stated that it was necessary for one of the patients, Mrs. Boon, to receive 5mg of Risperidone at meal time and administration should begin that day. At the next meal time Natalie gave 5mg of Risperidone to Mrs. Boon.

*This next section will deal with questions of Conformity.*
 *Instructions:* Conformity is the matching of attitudes, beliefs or behaviours to group norms. The different groups that we may conform to could include society, family, our class, our friends, or even a crowd at a sporting event or shopping center.

Examples of Conformity would be the situations such as those described for Jane, where she gave the same answer on an anatomical question as her classmates, or Steven, where he gave a Respiratory Rate and Heart Rate close to the ones given by his classmates.
These are only examples and you may think of very different experiences.
  
As you think about the questions below we would like you to think about the questions in terms of your education and professional development as a health professional. Within this guideline the situations you think of can be diverse and varying, the settings could range from a classroom experience to a patient care setting such as in a clinic. 
Many health professionals have reported experiencing these kinds of situations

1. Please estimate in the past **WEEK** how many incidences you can recall where **YOU** acted based on how one or more other people behaved?

|  | 0 | 1 | 2 | 3 | 4 | 5 | 6 | 7 | 8 | 9 | 10 |
| --- | --- | --- | --- | --- | --- | --- | --- | --- | --- | --- | --- |

| Please Indicate (1) | 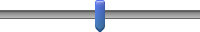 |
| --- | --- |

2.  Please estimate in the past **WEEK** how many incidences you can recall where you observed a **PEER** acting based on how one or more other people behaved?

|  | 0 | 1 | 2 | 3 | 4 | 5 | 6 | 7 | 8 | 9 | 10 |
| --- | --- | --- | --- | --- | --- | --- | --- | --- | --- | --- | --- |

| Please Indicate (1) | 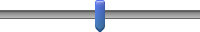 |
| --- | --- |

3. Please estimate in the past **MONTH**how many incidences you can recall where **YOU** acted based on how one or more other people behaved?

|  | 0 | 1 | 2 | 3 | 4 | 5 | 6 | 7 | 8 | 9 | 10 | 11 | 12 | 13 | 14 | 15 | 16 | 17 | 18 | 19 | 20 |
| --- | --- | --- | --- | --- | --- | --- | --- | --- | --- | --- | --- | --- | --- | --- | --- | --- | --- | --- | --- | --- | --- |

| Please Indicate (1) | 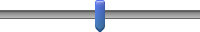 |
| --- | --- |

4.  Please estimate in the past **MONTH**how many incidences you can recall recall where you observed a **PEER** acting based on how one or more other people behaved?

|  | 0 | 1 | 2 | 3 | 4 | 5 | 6 | 7 | 8 | 9 | 10 | 11 | 12 | 13 | 14 | 15 | 16 | 17 | 18 | 19 | 20 |
| --- | --- | --- | --- | --- | --- | --- | --- | --- | --- | --- | --- | --- | --- | --- | --- | --- | --- | --- | --- | --- | --- |

| Please Indicate (1) | 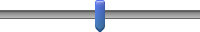 |
| --- | --- |

5. Please estimate in the past **SIX MONTHS** how many incidences you can recall where **YOU** acted based on how one or more other people behaved?

|  | 0 | 5 | 10 | 15 | 20 | 25 | 30 | 35 | 40 | 45 | 50 |
| --- | --- | --- | --- | --- | --- | --- | --- | --- | --- | --- | --- |

| Please Indicate (1) | 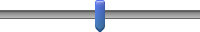 |
| --- | --- |

6. Please estimate in the past **SIX MONTHS**how many incidences you can recall where you observed a **PEER** acting based on how one or more other people behaved?

|  | 0 | 5 | 10 | 15 | 20 | 25 | 30 | 35 | 40 | 45 | 50 |
| --- | --- | --- | --- | --- | --- | --- | --- | --- | --- | --- | --- |

| Please Indicate (1) | 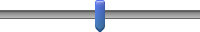 |
| --- | --- |

Next we will ask you some questions about your experiences with conformity.

1.  Have **YOU** ever carried out a task without being certain of the proper method yourself because you saw a peer do it that way?

- Yes (1)
- No (2)

2 .To what extent do you feel that **YOU** are “going with the crowd” when you need to perform a procedure or technique?

|  | Never (1) | Sometimes (2) | Often (3) | Most of the time (4) |
| --- | --- | --- | --- | --- |
| Please Indicate (1) |  |  |  |  |

3. Have you ever witnessed a **PEER** carry out a task because other people did it that way but you did not think that it was the correct method to use?

- Yes (1)
- No (2)

4.To what extent do you feel that your **PEERS** are “going with the crowd” when they need to perform a procedure or technique?

|  | Never (1) | Sometimes (2) | Often (3) | Most of the time (4) |
| --- | --- | --- | --- | --- |
| Please Indicate (1) |  |  |  |  |

5. Do **YOU** ever feel you need to alter your behaviour to align with the behaviour of those around you?

- Yes (1)
- No (2)

6. Do **YOU** ever feel that you need to alter your thinking to align with the thinking of those around you?

- Yes (1)
- No (2)

For the final questions about Conformity we will ask you about some of your perceptions of Conformity.

1. In your education to what extent do **YOU** feel that you need to “fake it until you make it”?

|  | Never (1) | Sometimes (2) | Often (3) | Most of the time (4) |
| --- | --- | --- | --- | --- |
| Please Indicate (1) |  |  |  |  |

2. Are **YOU** ever concerned with how your peers or teachers view you professionally?

|  | Never (1) | Sometimes (2) | Often (3) | Most of the time (4) |
| --- | --- | --- | --- | --- |
| Please Indicate (1) |  |  |  |  |

3.To what extent do **YOU** feel confident in your knowledge even when a peer may disagree with you?

|  | Not at all (1) | Very Little (2) | Somewhat (3) | Completely (4) |
| --- | --- | --- | --- | --- |
| Please Indicate (1) |  |  |  |  |

4.To what extent do **YOU** wish to be viewed as a typical member of your profession?

|  | Not at all (1) | Somewhat (2) | Highly (3) | Completely (4) |
| --- | --- | --- | --- | --- |
| Please Indicate (1) |  |  |  |  |

*This next section will deal with questions of Obedience*

*Instructions:*

Obedience to authority is defined as being compliant with an order, request, direction or instruction from a person who holds a position of authority over you. Often people in positions of authority can be thought of as those who are above us in a hierarchical structure. A person’s authority may come from formal or informal, legitimate or illegitimate means. A person in a formal position of authority may be a professor or a police officer. A person in an informal position of authority may be a parent or person a year or two ahead of you in your program.

Examples of Obedience to authority would be situations such as those described for Tom, where he “burst the blister”, or for Natalie, where she administered medicine after receiving instructions from Dr. Campbell. These are only examples and you may think of very different experiences.   As you think about the questions below we would like you to think about the questions in terms of your education as a health professional. Within this guideline the situations can be diverse and varying, the settings could range from a classroom experience to a patient care setting such as in a clinic. Many health professionals have reported experiencing these kinds of situations.

1.  Please estimate in the past **WEEK** how many incidences you can recall where **YOU** acted on the instructions of an authority figure that you did not believe was correct?

|  | 0 | 1 | 2 | 3 | 4 | 5 | 6 | 7 | 8 | 9 | 10 |
| --- | --- | --- | --- | --- | --- | --- | --- | --- | --- | --- | --- |

| Please Indicate (1) | 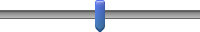 |
| --- | --- |

2. Please estimate in the past **WEEK** how many incidences you can recall where you observed a **PEER** acting on the instructions of an authority figure that you did not believe was correct?

|  | 0 | 1 | 2 | 3 | 4 | 5 | 6 | 7 | 8 | 9 | 10 |
| --- | --- | --- | --- | --- | --- | --- | --- | --- | --- | --- | --- |

| Please Indicate (1) | 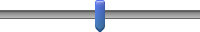 |
| --- | --- |

3. Please estimate in the past **MONTH** how many incidences you can recall where **YOU** acted on the instructions of an authority figure that you did not believe was correct?

|  | 0 | 1 | 2 | 3 | 4 | 5 | 6 | 7 | 8 | 9 | 10 | 11 | 12 | 13 | 14 | 15 | 16 | 17 | 18 | 19 | 20 |
| --- | --- | --- | --- | --- | --- | --- | --- | --- | --- | --- | --- | --- | --- | --- | --- | --- | --- | --- | --- | --- | --- |

| Please Indicate (1) | 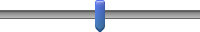 |
| --- | --- |

4. Please estimate in the past MONTH how many incidences you can recall where you observed a **PEER** acting on the instructions of an authority figure that you did not believe was correct?

|  | 0 | 1 | 2 | 3 | 4 | 5 | 6 | 7 | 8 | 9 | 10 | 11 | 12 | 13 | 14 | 15 | 16 | 17 | 18 | 19 | 20 |
| --- | --- | --- | --- | --- | --- | --- | --- | --- | --- | --- | --- | --- | --- | --- | --- | --- | --- | --- | --- | --- | --- |

| Please Indicate (1) | 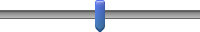 |
| --- | --- |

5.  Please estimate in the past SIX MONTHS how many incidences you can recall where **YOU** acted on the instructions of an authority figure that you did not believe was correct?

|  | 0 | 5 | 10 | 15 | 20 | 25 | 30 | 35 | 40 | 45 | 50 |
| --- | --- | --- | --- | --- | --- | --- | --- | --- | --- | --- | --- |

| Please Indicate (1) | 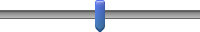 |
| --- | --- |

6. Please estimate in the past SIX MONTHS how many incidences you can recall  where you observed a **PEER** acting on the instructions of an authority figure that you did not believe was correct?

|  | 0 | 5 | 10 | 15 | 20 | 25 | 30 | 35 | 40 | 45 | 50 |
| --- | --- | --- | --- | --- | --- | --- | --- | --- | --- | --- | --- |

| Please Indicate (1) | 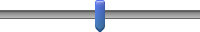 |
| --- | --- |

Next we will ask you some questions about your experiences with obedience.

1.  Have **YOU** ever felt you were in a situation where you could not contradict a person in a position of authority even though you believed the person in the position of authority was incorrect?

- Yes (1)
- No (2)

2.  Have **YOU** ever been subjected to negative consequences because you spoke up when you believed a person in a position of authority was incorrect?

- Yes (1)
- No (2)

3. Has a **PEER** ever expressed to you that they felt that they could not contradict a person in a position of authority even though they believed the person in the position of authority was incorrect?

- Yes (1)
- No (2)

4. Have you ever witnessed a **PEER** being subjected to negative consequences because they spoke up when they believed a person in a position of authority was incorrect?

- Yes (1)
- No (2)

5. Have **YOU** ever experienced any feelings of distress because you acted on the instructions from an authority figure that you did not believe were correct?

- Yes (1)
- No (2)

Skip To: Q47 If 5. Have YOU ever experienced any feelings of distress because you acted on the instructions from... = No

6.  If **YOU** have ever acted on the instructions from an authority figure that you did not believe were correct, how distressed did it make you feel?

|  | Not at all Distressed (1) | Moderately Distressed (2) | Highly Distressed (3) | Extremely Distressed (4) |
| --- | --- | --- | --- | --- |
| Please Indicate (1) |  |  |  |  |

7. Have you ever witnessed a **PEER** experience or display distress because they acted on the instructions of an authority figure that **THEY** did not believe were correct?

- Yes (1)
- No (2)

Skip To: End of Block If 7. Have you ever witnessed a PEER experience or display distress because they acted on the instru... = No

8. If you have witnessed a **PEER** experience or display distress because they acted on the instructions of an authority figure, in your opinion, how distressed did they appear?

|  | Not at all Distressed (1) | Moderately Distressed (2) | Highly Disstressed (3) | Extremely Distressed (4) |
| --- | --- | --- | --- | --- |
| Please Indicate (1) |  |  |  |  |

For the final questions about Obedience we will ask you about some of your perceptions of Obedience.

1. Have **YOU** ever acted on what you believed were the incorrect instructions of an authority figure because you were concerned with being perceived as competent?

- Yes (1)
- No (2)

2. Have **YOU** ever acted on what you believed were the incorrect instructions of an authority figure because you were concerned with how the authority figure may view you professionally?

- Yes (1)
- No (2)

3. Have **YOU** ever followed the actions of another person, or several other people, who were acting based on the instruction of an authority figure when you were not sure if what they were doing was correct?

- Yes (1)
- No (2)

4. Have **YOU** ever followed the instructions of an authority figure because you did not believe that you would be held responsible for the outcomes of those actions?

- Yes (1)
- No (2)

Finally we would like to give you some space to talk about any experiences, thoughts, or ideas you have about Conformity and Obedience in your education and professional life. You can write about these in the space below.

________________________________________________________________

Please indicate your age

|  | 0 | 10 | 20 | 30 | 40 | 50 | 60 | 70 | 80 | 90 | 100 |
| --- | --- | --- | --- | --- | --- | --- | --- | --- | --- | --- | --- |

| Please Indicate (1) | 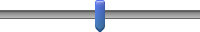 |
| --- | --- |

Please indicate your biological sex

- Male
- Female
- Other
- Prefer not to indicate

Please indicate your faculty

- Medicine and Dentistry (1)
- Nursing (2)
- Rehabilitation Medicine (3)
- Pharmacy and Pharmaceutical Sciences (4)
- Agricultural, Life & Environmental Sciences (5)
- Kinesiology Sport and Recreation (6)

Please indicate your program area

________________________________________________________________

Please indicate your program year

|  | 0 | 1 | 2 | 3 | 4 | 5 | 6 | 7 | 8 | 9 | 10 |
| --- | --- | --- | --- | --- | --- | --- | --- | --- | --- | --- | --- |

| Please Indicate (1) | 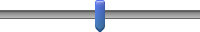 |
| --- | --- |
